# Extracorporeal gas exchange: when to start and how to end?

**DOI:** 10.1186/s13054-019-2437-2

**Published:** 2019-06-14

**Authors:** L. Gattinoni, F. Vassalli, F. Romitti, F. Vasques, I. Pasticci, E. Duscio, M. Quintel

**Affiliations:** 1 Department of Anesthesiology, Emergency and Intensive Care Medicine, University of Göttingen (UMG), Robert-Koch-Straße 40, 37075 Göttingen, Germany; 2 Department of Adult Critical Care, Guy’s and St Thomas’ NHS Foundation Trust, London, UK

## Introduction

In the last decade, primarily following the H1N1 pandemics [1], the extracorporeal respiratory assist is increasingly used [2, 3]. The acronym “ECMO”, i.e., ExtraCorporeal Membrane Oxygenation, is, however, somehow misleading as the artificial extracorporeal assist may affect both oxygenation and CO_2_ removal, as well as the hemodynamics, depending on how it is applied. In this commentary, we will limit our discussion to the respiratory extracorporeal support in veno-venous mode, primarily discussing the aspects, which are usually under-evaluated.

### Various options for extracorporeal support

Table 1 was first published more than 40 years ago [4] and summarizes the main characteristics and options through which the extracorporeal support may be applied. As shown, all the possible application were foreseen and most of them actually tested in the following years. As shown, two main features characterize the extracorporeal support: cannulation (veno-venous vs veno-arterial) and extracorporeal blood flow.

- In the veno-venous configuration, the artificial and the natural lung are connected in series, as the blood flow entering the membrane lung is re-directed into the natural lung, after the artificial gas exchange. The hemodynamics are not affected by this configuration, which works solely as a respiratory support. In contrast, in the veno-arterial configuration, the artificial and the natural lung are arranged in parallel: the flow leaving the artificial lung is diverted in the arterial section and the natural lung is proportionally under-perfused. The greatest difference between veno-venous and veno-arterial approach is not related to the gas exchange, as the amount of oxygen transferred and CO_2_ removed are exactly the same (if the operating conditions of the membrane lung are the same), but to the hemodynamic impact, as the veno-arterial configuration provides both respiratory and cardiac support.

- The second feature is the amount of blood flow and gas flow used to ventilate the artificial lung: to oxygenate venous blood entering the membrane lung, the gas flow required equals the oxygen sufficient to fully saturate the hemoglobin passing through the artificial lung. As an example, if 1 l of venous blood with10 g/dL of hemoglobin and saturation 70% enters the membrane lung every minute, a transfer of 42 ml of 100% oxygen per minute from the gas compartment of the membrane lung would be sufficient to fully saturate the blood leaving the membrane lung. Therefore, being the possibility to “charge” oxygen limited by the hemoglobin concentration and its saturation in the venous blood, the oxygen transfer to the membrane lung is primarily function of the extracorporeal blood flow. In the previous example, 4 l of extracorporeal blood flow, in the absence of re-circulation, would provide fully saturated blood with a gas flow into the membrane lung of only 168 ml/min. All the gas is absorbed, and no gas leaves the membrane lung

**Table 1:** Comparative technical difficulty of hemodialysis, extracorporeal removal of carbon dioxide, and extracorporeal oxygenation

	Renal hemodialysis	Extracorporeal removal of carbon dioxide	Extracorporeal oxygenation
Extracorporeal blood flow (ml/min)	200–300	500–1000	2000–4000
Blood pumping	optional	optional	required
Hemodynamic changes	small	small	major
Vascular access	A-V shunt or A-V fistula	A-V shunt or A-V fistula or V-V pumping	V-A or V-V
Surgical complexity	simple	simple	complex
Complexity of equipment	moderate	simple	advanced
Requirement for heparin	small	small	large

Table 1 Reproduced with permission from Gattinoni et al., Control of intermittent positive pressure breathing (IPPB) by extracorporeal removal of carbon dioxide, British Journal of Anesthesia, © 1978 Elsevier Inc. [4]

The CO_2_ transfer, due to the physicochemical characteristics of CO_2_ in the blood, follows a complete different scheme. The CO_2_ content in the blood is primarily function of the strong ion difference: for the same PCO_2_, the CO_2_ content depends on the difference between expected and actual strong ion difference (i.e., the base excess). As an example, at a base excess of − 10 mEq/L and PCO_2_ 40 mmHg compared to a base excess of 0 mEq/L at the same PCO_2_, the total amount of CO_2_ in the blood (dissolved + bicarbonate + carbo-amino compounds) goes from 37 to 50 ml/dL. In normal conditions, with pH close to 7.4 and PCO_2_ in the range of 40–50 mmHg, the amount of total CO_2_ in the blood is roughly 1 ml per mmHg of CO_2_, i.e., with a PCO_2_ of 45 mmHg and base excess 0 mEq/L (strong ion difference 42 mEq/L), the CO_2_ content is about 45 mL/dL. This means that the near total metabolic production of CO_2_ is equivalent to the CO_2_ present in about 500 ml of blood. Therefore, if the blood flowing through the membrane lung is ventilated at a very high rate, the total metabolic CO_2_ production may be cleared from an amount of blood similar with the one used during continuous veno-venous hemofiltration.

Therefore, to provide 200 ml/min of oxygen, high extracorporeal blood flow is required, with minimum ventilation of the artificial lung, while the same amount of CO_2_ may be cleared for less than one fourth of the blood flow, but very high ventilation is required. The physiology of the gas exchange with the artificial lung clearly indicates that the oxygenation and CO_2_ removal function may be easily dissociated in the artificial extracorporeal system, and this accounts for the tremendous possibility of intervention which is possible using the artificial lung systems.

### Rationale

The veno-venous extracorporeal support, through different settings, recognizes two primary rationales:

- Rescue intervention for tissue hypoxia, primarily due to respiratory failure (high-flow veno-venous ECMO) [5]
- Reduction of mechanical ventilation and related damages in ARDS [6–8], status asthmaticus [9, 10], and COPD exacerbation (low-flow ECCO_2_R or minimally invasive ECCO_2_R) [11, 12]. To this, another possible use of minimally invasive ECCO_2_R may be considered for COPD patients in order to improve the quality of life by programmed CO2 dialysis [13]

### Rescue high-flow V-V ECMO

The rescue applies when hypoxemia is per se “life-threatening”. Obviously, this condition cannot be defined neither by a single value of PaO2, nor by a combination of more variables (e.g., hypoxemia and hypercapnia). Indeed, the life-threatening hypoxemia is a clinical judgment, which accounts for age, comorbidities, pathophysiological alterations, and time course of the disease of the patient. As far as we know, the PaO_2_ of 19 mmHg is the lowest level of arterial PO_2_ recorded in healthy living subjects on the Everest [14]; this values are the same recorded in turtles [15], penguins [16], and whales [17] during deep immersions; and, most interestingly, these are the normal values during human fetal life [18]. This stresses the nonsense of considering a single value of PO_2_ as life-threatening threshold, without considering the perfusion pattern. Indeed, it is common in ICU, during extracorporeal support, to observe occasionally patients without any relevant organ failure, but the lung, despite PaO_2_ as low as 30 mmHg if the hemodynamics are adequate. Therefore, we believe that the attending physician is the most qualified “measuring tool” to detect hypoxemic life-threatening conditions, as he/she may integrate the myriad of information beyond PaO_2_ levels, posing the patient at immediate risk of dying. In reality, the bulk of studies dealing with ECMO, since the first randomized controlled trial by Warren Zapol in the middle of 1970s [19], used the hypoxemia threshold as entry criteria for high-flow ECMO (see Table 2). Of note, the most recent ECMO study, i.e., EOLIA trial [20], used criteria not very different from those used four decades before and provided a strong signal that ECMO, used as a rescue therapy of severe hypoxemia, may lead to survival benefits.

**Table 2:** Entry criteria of extracorporeal support trials

Study	Patients enrolled	Inclusion criteria
NIH adult ECMO trial Zapol et al. 1979, JAMA	90	Severe ARF: -PaO_2_ < 50 mmHg for at least 2 h despite 100% FIO_2_ and 5 cmH_2_O of PEEP (fast entry) -PaO_2_ < 50 mmHg for at least 12 h despite 60% FIO_2_ and 5 cmH_2_O of PEEP or a Qs/Qt > 30% with 100% of FIO_2_ and 5 cmH_2_O PEEP
PCIRV vs ECCO2R Morris, 1994, Am J Respir Crit Care Med	40	-ARDS (defined as P_(a/A)_O2 < 0.2, bilateral chest radiographic infiltrates, total compliance < 50 ml/cmH_2_O, wedge pressure < 15 mmHg and no signs of heart failure)
		-ECMO criteria: - PaO_2_ < 50 mmHg for at least 2 h despite 100% FIO_2_ and 5 cmH_2_O of PEEP (fast entry) - PaO_2_ < 50 mmHg or Qs/Qt > 30% for at least 12 h despite 60% FIO_2_ and 5 cmH_2_O of PEEP, in a > 48 h ICU patients (slow entry)
CESAR trial Peek et al. 2009, Lancet	180	-Severe but potentially reversible respiratory failure (Murray score > 2.5 or hypercapnia with arterial pH < 7.2)
		-Age 18–65
		-Ventilation/high FIO_2_ < 7 days
		-No cranial bleeding
		-No contraindication to heparin
		-No contraindication to continuation of the active treatment
EOLIA trial Combes et al. 2018, NEJM	249	-ARDS
		-Mechanical ventilation < 7 days
		-With (despite ventilator optimization): • PaO_2_/FIO_2_ < 50 for at least 3 h • PaO_2_/FIO_2_ < 80 for at least 6 h • Arterial pH < 7 .25 with PaCO_2_ > 60 mmHg for at least 6 h

### Low-flow extracorporeal CO_2_ removal

The definition of low flow is absolutely arbitrary, as it may range from 300 to 400 ml/min up to 1000–1500 ml/min. In this range of flow, the clearing of CO_2_ relative to the metabolic production may range from 20 up to 100% depending on input CO_2_, membrane lung surface, and sweep gas flow [21]. The main difference between low and high-flow extracorporeal support, in our opinion, is that the contribution to the oxygenation is limited at low flow, i.e., not higher than 30% at 1500 ml/min of extracorporeal blood flow and negligible at 300–400 ml/min. The concept of extracorporeal CO_2_ removal was introduced by Kolobow when the dismal results of the Zapol’s trial were informally known (90% mortality in control and ECMO groups). The initial input for extracorporeal CO_2_ removal by Kolobow was to explore the possibility of CO_2_ dialysis in COPD patients, aiming at quality of life improvement. For this purpose, he developed a special artificial lung with high surface and thin membrane (the carbon dioxide membrane lung, CDML) to maximize CO_2_ removal [22]; however, when testing the performances of the CDML, we found that removing CO_2_ in healthy spontaneously breathing sheep allowed a complete control of their ventilation [6]. Indeed, if 50% of CO_2_ produced by an animal in 1 min is removed through the artificial lung, the animal reset its own ventilation by decreasing alveolar ventilation by 50%, at constant PaCO_2_. This observation led to the idea of using the extracorporeal CO_2_ removal to decrease the impact of high pressure/volume ventilation, which was the rule at that time in ARDS patients. The idea of CO_2_ dialysis was abandoned in favor of the idea of “lung rest” in severe ARDS [4, 23]. These physiological principles are still valid today and provide a basis for introducing a “gentle” ventilation in ARDS.

Due to these premises, the indication to apply ECCO_2_R as a tool to decrease the harms of mechanical ventilation should be based on a hypothetical threshold, defining the risk of unacceptable ventilation-induced lung injury (VILI). Unfortunately, as far as we know, this approach has never been used and also for ECCO_2_R the indications are based on the impairment of oxygenation. In the last few years, we tried to identify a comprehensive variable to estimate the risk of VILI, i.e., the mechanical power, which accounts for excessive tidal volume, excessive driving pressure, respiratory rate, inspiratory flow, and PEEP. This approach led to consistent result in experimental animals and appears promising when the mechanical power has been tested in large ARDS population [24–26].

### Extracorporeal support: when to start

#### High-flow veno-venous ECMO

The main drive to begin the high-flow extracorporeal support in ARDS patient is hypoxemia, when its level is considered as “life-threatening” [27]. As shown in Table 2 in which we summarize the entry criteria of the larger randomized trials, the PaO_2_/FIO_2_ used to apply the extracorporeal support is always below 100, a level which was used to define the refractory hypoxemia since the first description of ARDS [28], indicating that even 100% FIO_2_ was insufficient to restore normal oxygen tension in the arterial blood. Undoubtedly, the primary indication for V-V ECMO remains the hypoxemia. We may wonder, however, if a real threshold for hypoxemia exists, as the patient with different biological resources, comorbidities, and hemodynamics may present different “adequate” PaO_2_. In addition, the same PaO_2_/FIO_2_ threshold below 100 may encompass different shunt fractions depending on several factors [29]. Therefore, it is not surprising (and luckily it is the best solution) that, in clinical practice, are the attending physicians, usually in team, to decide if that particular hypoxemia in a given patient is such as to require the membrane lung application, considering its values together with a myriad of other anamnestic and pathological information.

The use of high-flow V-V ECMO, as a rescue for life-threatening hypoxemia, has never been questioned. Few noted that paradoxically the PaO_2_ in control and ECMO patients is the same throughout the clinical course, as clearly shown in EOLIA trial. Therefore, we may wonder if the use of high flow is really necessary in patients with adequate hemodynamics. To rationally answer this question, the mechanisms of oxygenation during high-flow V-V ECMO must be discussed. Let us assume that in a patient, in whom 4–5 L/min of extracorporeal blood flow are applied, the amount of oxygen transfer per minute is close to the total oxygen consumption (200–300 ml/min). This has two major consequences:

- The oxygen transfer in the natural lung decreases proportionally to the increase of oxygen saturation of hemoglobin perfusing the open lung units. Indeed, the PO_2_ in the pulmonary capillaries, perfusing the open lung units, only depends on FIO_2_, barometric pressure, and respiratory quotient. Therefore, the drive for the oxygen transfer in the natural lung is the difference between PAO_2_ (equal to the pulmonary capillary partial pressure) and the PVO_2_/saturation of the blood entering the venous side. Higher PvO_2_ and oxygen saturation implies decrease of oxygen transfer. It is worth to understand that the capillary PO_2_ of the ideally perfused pulmonary unit does not change, whatever is the ECMO blood flow.

**Fig. 1:**
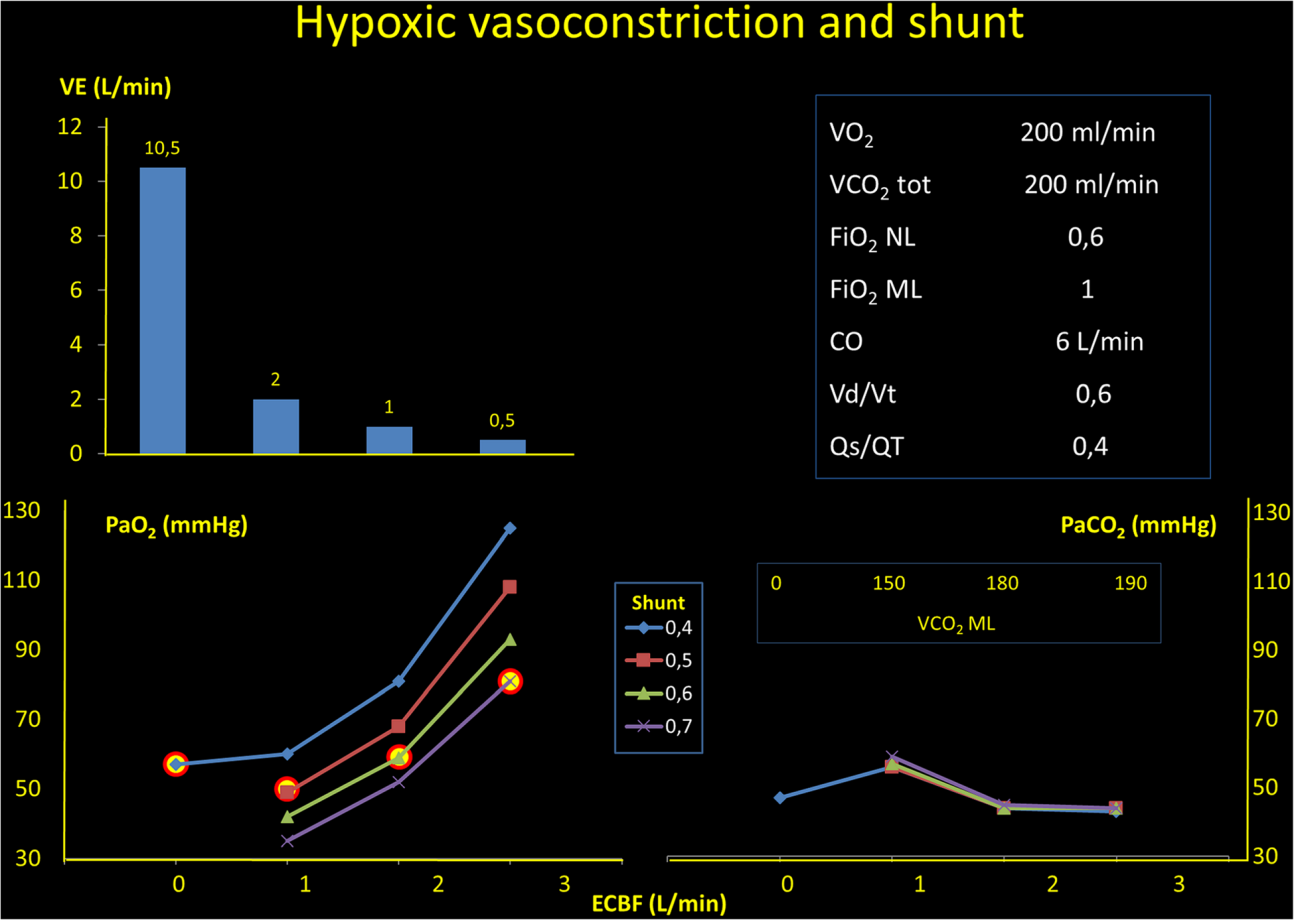
In the right square, we present the starting conditions of this analysis. In the left upper panel, we present the decrease of the total ventilation to maintain an unchanged PCO_2_ (lower right panel) when the extracorporeal blood flow is increased. In the left lower panel, we show which would be the arterial PO_2_ as a function of the extracorporeal blood flow, if the shunt fraction would be unmodified. As shown, an extracorporeal blood flow of 1.5 L/min, if the shunt increases to 0.4 to 0.7 (a common finding in this condition), the PaO_2_ increase, if any, is negligible

Second, the increased oxygen content in the venous side increases the hemoglobin oxygen saturation in the pulmonary artery and decreases the hypoxic vasoconstriction, which, although dampened, is well-presented and effective in ARDS patients [30]. Indeed, the hypoxic vasoconstriction depends both on alveolar hypoxia, unlike in ARDS patients ventilated with high FIO_2_, and on oxygen partial pressure in the mixed venous blood [31, 32]. When the saturation of the blood perfusing the gasless regions increases, the fraction of blood flowing through them increases remarkably up to 60–70%. This explains why the PaO_2_, in high veno-venous blood flow, at the beginning of extracorporeal support, does not increase dramatically, but only by few mmHg. Indeed, the oxygenation gain in the arterial side, therefore, is only due to the increased oxygen saturation of the blood flowing through the shunted area, which increases due to the release of hypoxic vasoconstriction. In Fig. 1, this phenomenon is quantitatively exemplified.

### Low-flow ECMO: when to start

Low-flow ECMO is a tool to allow the decrease of the possible damage of mechanical ventilation in the baby lung, by reducing minute ventilation, while maintaining normal CO_2_. As the harm of mechanical ventilation derives from unphysiological stress and strain repeated over time up to the near-total lung capacity of the baby lung, the rationale indication for extracorporeal support should be based on thresholds derived from lung mechanics. As far as we know, however, this approach has never been used and the primary criteria for ECCO_2_R application are similar to the ones used for high-flow ECMO, i.e., hypoxemia. Interestingly, even the recently proposed trial that combines low-flow extracorporeal CO_2_ removal and ultra-protective lung strategy indicates as entry criteria the presence of moderate ARDS, based on oxygenation criteria [33].

We recently proposed the mechanical power as a unifying variable to select the harmful threshold of mechanical ventilation [34]: indeed, mechanical power includes tidal volume, driving pressure [35], respiratory rate [36], flow [37], and positive end-expiratory pressure [38]. Each one has been shown, isolated or in association, to cause ventilator-induced lung injury. In experimental animals of middle size, a possible threshold around 13 J/min discriminates between major and relatively minor ventilator damage and we are trying to investigate a possible threshold in human beings. Nowadays, the minimally invasive ECCO_2_R is primarily suggested for the treatment of COPD exacerbation, while in severe ARDS the technique is not considered, due to low impact on blood oxygenation.

Recently, we found that a relevant amount of CO_2_ may be cleared in a minute with an extracorporeal blood flow not greater than 400 ml/min, when adequate membrane lung surface and sweep gas flow are used [21]. Depending on the metabolic CO_2_ production, this set may provide from 50% to near 100% of metabolic CO_2_ clearing, while the oxygen added artificially is not greater than 10–20 ml/min. If we consider that the severe ARDS patients, treated with high-flow ECMO, are still ventilated with minute ventilation ranging from 6 to 10 L/min, we may wonder if such effect cannot be reached with the minimally invasive approach instead of high-flow ECMO. Indeed, in hemodynamically stable patients, even low oxygenation (circa 50 mmHg) can be well tolerated if the hypoxic vasoconstriction is maintained, while the modification of mechanical ventilation could be similarly reduced. It is possible that, in the near future, the actual difference between ECMO and ECCO_2_R in severe ARDS will be reconsidered under the light of these pathophysiological mechanisms.

### Extracorporeal support: when to stop

The logical indication to stop either ECMO or ECCO_2_R should be the cessation of the condition for which ECMO or ECCO_2_R have been instituted. Therefore, the condition for stopping high-flow ECMO would be the maintenance of adequate oxygenation without extracorporeal support and, for ECCO_2_R, the mechanical ventilation below any harmful threshold. In practice, the approach used in the clinical practice to remove the extracorporeal support is more pragmatic than rational. Actually, the “weaning process” starts from the beginning of the extracorporeal support by progressively reducing the possible harmful component of mechanical ventilation (FIO_2_ and pressures). Indeed, during full blown ARDS, the severely hypoxemic patients at the beginning are kept sedated/paralyzed with relatively high mean airway pressure, while the minute ventilation is reduced at different extent. During this phase, any attempt of a spontaneous breathing may be ineffective as the respiratory drive of the patient, independently of normal blood gases, is so high that the spontaneous breathing would be more dangerous than whatever mechanical ventilation applied [39]. However, when the disease leading to ARDS is under control, the respiratory drive tends to normalize. The steps of weaning relate first to progressive decrease of FIO_2_ down to 40–50%, then to decrease of PEEP (1–2 cmH_2_O/h). The steps are interrupted if the oxygenation deteriorates. When it is possible to maintain oxygenation with circa 40% oxygen and circa 10 cm H_2_O of PEEP, the patients are usually ready for disconnection. Nowadays, at this stage, we test the patient capability to breathe spontaneously and/or to tolerate pressure support ventilation by a stepwise decrease of the sweep gas flow, while measuring at the same time the esophageal pressure swings. If the negative swings of esophageal pressure are < 15 cm H_2_O at a respiratory rate < 30 rpm, the patient is decannulated. This is one of the several possible ways, which are anyway based on the achievement of two targets: adequate oxygenation and arterial PCO2 during safe spontaneous/mechanical ventilation.
